# *Capsicum annuum* TC48 shows both resistance and tolerance to cucumber mosaic virus and is a potential genetic resource for pepper improvement

**DOI:** 10.1016/j.abiote.2026.100081

**Published:** 2026-07-29

**Authors:** Tonghui Li, Xi Wang, Xueyan Yao, Ye Liu, Manru Song, Yingjie Mi, Hongji Yang, Lulu Liu, Enguang Li, Junyue Sun, Hailong Yu, Nir Ohad, Xuan Huang, Feng Liu, Yan Xue

**Affiliations:** a State Key Laboratory of Wheat Improvement, Peking University Institute of Advanced Agricultural Sciences, Shandong Laboratory of Advanced Agricultural Sciences in Weifang, Weifang, 261325, China; b School of Plant Science and Food Security, Tel Aviv University, Tel Aviv, 69978, Israel; c Engineering Research Center for Germplasm Innovation and New Varieties Breeding of Horticultural Crops, Key Laboratory for Vegetable Biology of Hunan Province, College of Horticulture, Hunan Agricultural University, Changsha, 410128, China; d Provincial Key Laboratory of Biotechnology of Shaanxi, Key Laboratory of Resource Biology and Biotechnology in Western China, Ministry of Education, College of Life Sciences, Northwest University, Xi’an, 710069, China; e Institute of International Rivers and Eco-security, Yunnan University, Kunming, 650500, China; f College of Life Sciences, Shandong Normal University, Jinan, 250358, China; g Institute of Vegetables and Flowers, Chinese Academy of Agricultural Sciences, Beijing, 100081, China

Dear Editor,

Cucumber mosaic virus (CMV) infection in pepper (*Capsicum* spp.) causes systemic symptoms, such as leaf chlorosis, dwarfism, flower abscission, and fruit lesions, significantly reducing crop yields and market value. CMV infects over 1200 plant species from more than 100 families, making it a serious concern for agriculture. There are currently no effective chemicals that directly target CMV. In addition, the insecticides that affect CMV transmission vectors are environmentally unfriendly and expensive. A more sustainable strategy for CMV control is to develop resistant cultivars. However, despite intensive efforts, CMV resistance in commercial pepper lines remains insufficient, prompting the need for a better understanding of the molecular mechanisms underlying CMV responses in pepper.

To mitigate the detrimental effects of pathogen infection, plants have evolved strategies including both resistance (limiting pathogen spread) and tolerance (maintaining growth/fitness despite pathogen proliferation). Resistance against viral pathogens includes pattern-triggered immunity (PTI), effector-triggered immunity (ETI), and RNA interference (RNAi) [1,2]. PTI can be triggered by pathogen associated molecular patterns (PAMPs), damage associated molecular patterns (DAMPs), as well as dsRNAs derived from viral replication [2,3]. These highly conserved pathogen components are recognized by cell surface-localized pattern recognition receptors (PRRs). To escape PTI, pathogens have evolved strategies to directly secrete virulence effectors into the cytoplasm. In response, plants have developed resistance (R) proteins to detect effectors and initiate ETI [2]. The activation of PTI and ETI leads to a largely overlapping set of immune responses, including the activation of calcium signaling, production of reactive oxygen species, transcriptional reprogramming, and establishment of systemic acquired resistance (SAR), with ETI involving more intense responses than PTI [4]. RNAi is triggered by dsRNAs derived from DNA and RNA viruses. In *Arabidopsis thaliana*, DICER-LIKE (DCL) 4 and DCL2 act hierarchically to produce anti-viral small RNAs (sRNAs) [5,6]. These sRNAs are loaded onto ARGONAUTE (AGO) proteins that exert anti-viral activities [7]. As a counter-defensive strategy, some viruses have developed viral suppressor of RNAi (VSR) proteins. Well-characterized VSRs include P21 from western yellow virus, P19 from tomato bushy stunt virus (TBSV), and 2b from CMV. These proteins bind to and suppress the activities of sRNAs [[8], [9], [10], [11], [12]]. 2b also disrupts AGO1 function through a direct interaction [10].

Despite their effectiveness, coordinated immune responses may damage the host plant. The reallocation of energy from growth to defense often leads to reductions in biomass and yield. In addition, resistance imposes strong selective pressure on pathogens, driving the evolution of new, resistance-breaking pathovars. As an alternative, some plants have developed tolerance, i.e., the ability to maintain growth and fitness regardless of pathogen proliferation [13]. Instead of combating pathogens directly, disease-tolerant plants mitigate the damage caused by both pathogens and host immune responses [13]. Therefore, tolerant plants remain healthy despite pathogen proliferation and are unlikely to drive the evolution of resistance-breaking pathovars [13,14]. Under pathogen attack, tolerant plants may prioritize energy reallocation toward growth and the repair of damage caused by pathogens or host immunity [13]. In the tolerant potato (*Solanum tuberosum*)–PVY interaction, the transcription of photosynthetic genes is activated in systemic leaves during early infection. Intriguingly, this transcriptional change does not translate into protein accumulation, although how such transcriptional reprogramming is achieved remains elusive [15]. Despite the widespread occurrence of tolerance and its potential importance in agriculture, mechanisms underlying disease tolerance remain far less understood than those underlying resistance. Therefore, in this study, we characterized the *Capsicum annuum* line TC48, which is barely affected by CMV throughout vegetative and reproductive growth, and compared it to the CMV-susceptible line ST-8 (as a negative control) to explore the CMV tolerance mechanism (Supplementary Methods).

At 30 days post infection (dpi) with CMV, ST-8 developed obvious chlorosis and distortion of systemic leaves, while TC48 remained largely asymptomatic, suggesting that vegetative growth is unaffected in TC48 (Fig. 1A). To evaluate reproductive fitness, we collected fruits from individual plants. In TC48, fruit size and fruit weight after CMV infection were not significantly different from those of the mock control, whereas ST-8 displayed smaller fruits and a nearly 2-fold reduction in fruit weight (Fig. 1B and C). ST-8 also produced fewer seeds after CMV infection, whereas TC48 retained a similar number of seeds to the mock control (Fig. 1D and E). Thus, TC48 was barely affected by CMV during both vegetative and reproductive growth.

**Fig. 1:**
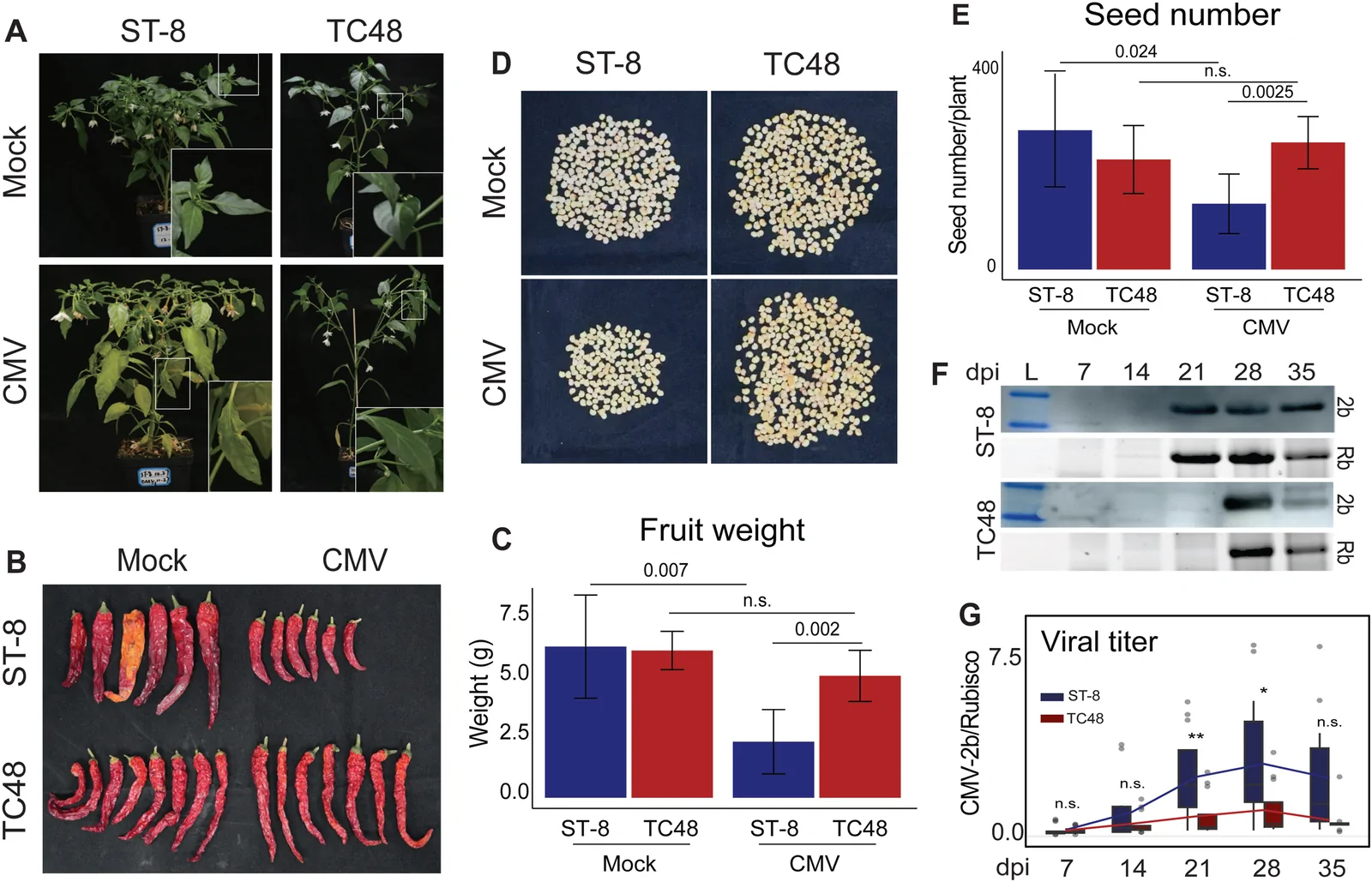
TC48 exhibits partial resistance and tolerance to CMV. **A** Phenotypes of ST-8 and TC48 plants 30 days post mock treatment or CMV inoculation. **B–C** Fruits harvested from ST-8 or TC48 individuals following mock treatment or CMV inoculation (B), and quantification of fruit weight (C). The error bars represent standard deviation (SD). Statistical significance was assessed using Student’s t-test. n.s. indicates no significant difference (*P* ≥ 0.05). **D-E** Seeds harvested from ST-8 or TC48 individuals following mock treatment or CMV inoculation (D), and quantification of seed number (E). **F** Immunoblots probed with anti-CMV 2b antibody from one representative ST-8 and one representative TC48 individual at 7 to 35 days post CMV inoculation. Data from four independent biological replicates were used for quantification, and results from one representative individual are shown. **G** Quantification of CMV 2b levels from four independent inoculations.

To investigate viral proliferation and movement, we measured CMV 2b protein levels in systemic leaves by immunoblotting using four independent biological replicates (Fig. 1F and G, Supplementary Methods). Despite individual variations, we consistently observed the following patterns: 1) No CMV 2b was detected at 7 dpi, and it was occasionally detected at low levels at 14 dpi. 2) At 21 or 28 dpi, CMV 2b reached similar levels between ST-8 and TC48, while TC48 individuals remained asymptomatic. 3) Overall, lower CMV 2b levels were detected in TC48, and fewer TC48 individuals accumulated high levels of CMV 2b systemically (Fig. 1F and G). 4) During late infection (after 50 dpi), CMV 2b often reached similar levels between ST-8 and TC48 (Fig. S1A). Patterns 2 and 4 indicate that TC48 is tolerant to CMV, whereas pattern 3 is consistent with partial resistance. Therefore, we defined TC48 as being partially resistant at early stages and tolerant at late stages of infection.

To explore the underlying molecular events, we performed RNA seq on systemic leaves at 7 dpi, when 2b was undetectable, and 30 dpi, when disease phenotypes were visible (Supplementary Methods). We first compared CMV- to mock-treated samples within the same genotype. Genes involved in defense responses were upregulated in ST-8 at 7 dpi, while genes involved in photosynthesis were downregulated (Fig. 2A, Table S1). This is consistent with the previous finding that photosynthesis is among the earliest processes affected by stress conditions and that resources are preferentially redirected from growth to defense responses [16]. Notably, in TC48 at 7 dpi, genes involved in photosynthesis were upregulated, together with genes involved in ATP synthesis, carbon fixation, and growth. Among the downregulated defense-related genes in TC48 at 7 dpi were several *1-AMINOCYCLOPROPANE-1-CARBOXYLATE OXIDASE* homologs as well as genes in the terpenoid biosynthetic pathway, suggesting that the ethylene pathway and the production of defensive secondary metabolites were likely downregulated in these plants (Fig. 2A, Table S1). These observations suggest that transcriptionally, TC48 prioritized growth over defense when challenged with CMV at 7 dpi. While similar phenomena have been reported previously for tolerant plant–pathogen interactions, the biological significance and underlying mechanisms remain largely unknown [13,15].

**Fig. 2:**
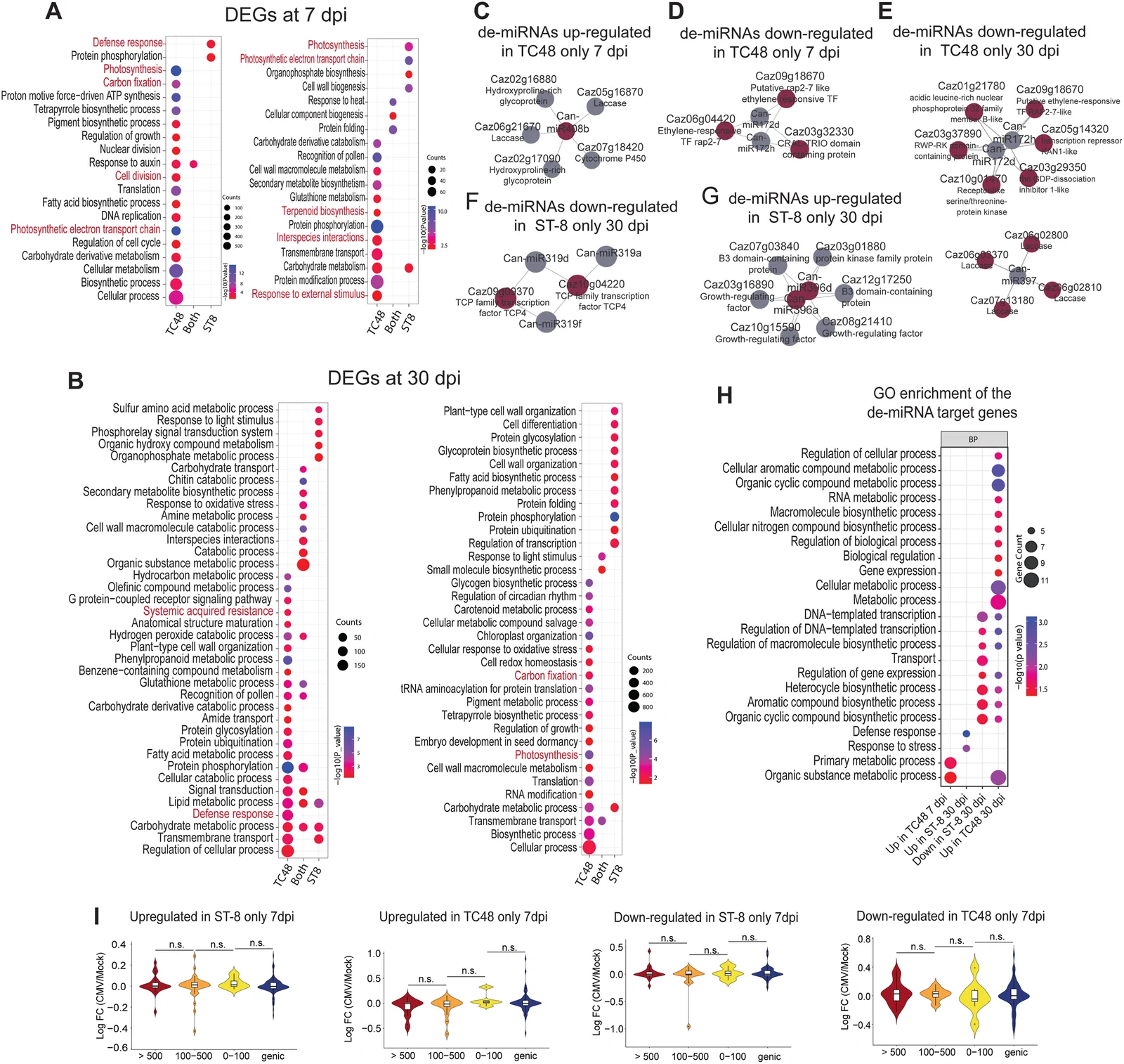
Transcriptional reprogramming underlying CMV resistance in TC48. **A-B** Enriched GO terms in the biological processes category of up- and downregulated genes in TC48 relative to ST-8 at 7 dpi (A) and 30 dpi (B) with CMV. **C-G** Interaction network of de-miRNAs with higher abundance in TC48 only (C) at 7 dpi, lower abundance in TC48 only (D) at 7 dpi, lower abundance in TC48 only (E) at 30 dpi, lower abundance in ST-8 only (F) at 30 dpi, and higher abundance in ST-8 only (G) at 30 dpi. **H** Enriched GO terms of the de-miRNA target genes in ST-8 and TC48 after CMV challenge. **I** Log FC of expression of CMV/Mock genes downstream of the de-siRNAs at 7 dpi with CMV. The width of each violin represents the kernel density of the data distribution. The embedded boxplots indicate the median and interquartile range (IQR). Statistical significance was determined using Student’s *t*-test. ns, not significant (*P* ≥ 0.05).

At 30 dpi, many genes encoding NB-ARC domain-containing proteins, putative late blight resistance protein, and MLO-like protein were activated in TC48, suggesting the large-scale upregulation of pathogen recognition systems and potentially ETI. We also observed the activation of SAR at this time point (Fig. 2B, Table S1). Together, these data point to a bimodal dynamic relationship between energy metabolism and defense responses in TC48 after CMV infection at the transcriptional level. Whether similar changes occur at the protein level remains to be tested. Notably, bimodal responses were also evident when we compared TC48 with ST-8 (Fig. S2B–D).

Considering the critical roles of sRNAs in plant–virus interactions, we focused on sRNAs, including 21- and 22-nt miRNAs as well as 24-nt siRNAs, to explore the regulatory mechanisms underlying this coordinated transcriptional reprogramming (Supplementary Methods). Overall, we detected more differentially expressed miRNAs (de-miRNAs) at 30 dpi compared to 7 dpi (Fig. S3A, Table S2). Among these, the abundance of miR408 increased specifically in TC48 at 7 dpi (Fig. 2C). Well-characterized mRNA targets of miR408 include laccase (LAC) genes [17], which play essential roles in lignin, jasmonic acid, flavonoid, and secondary cell wall biosynthesis in plants and are therefore important for defense against pathogens [17]. *A. thaliana* overexpressing *MIR408* exhibited morphological alterations, such as increased leaf area, petiole length, plant height, and overall biomass, whereas overexpressing the miR408 target *LAC13* had the opposite effects [17]. The increased miR408 abundance is consistent with our observation that growth was prioritized in TC48 during early infection.

Showing the opposite pattern, miR172 levels specifically decreased in TC48 at both 7 and 30 dpi (Fig. 2D and E). miR172 targets genes encoding ethylene responsive factors (ERFs), which regulate stress responses [18]. For instance, AtERF1 regulates the expression of *JASMONATE-RESPONSIVE PLANT DEFENSIN* (*PDF1.2*), *BASIC CHITINASE*, *RESPONSIVE TO DESICCATION29B* (*RD29B*), *EARLY RESPONSE TO DEHYDRATION7* (*ERD7*), and *RD20*, thereby conferring biotic and abiotic stress resistance in Arabidopsis [19,20]. Notably, miR172 levels were reduced at both 7 and 30 dpi in TC48, whereas genes involved in defense responses were down- and upregulated, respectively, at these time points. As mentioned above, several *1-AMINOCYCLOPROPANE-1-CARBOXYLATE OXIDASE* homologs were repressed at 7 dpi in TC48, pointing to the transcriptional downregulation of ethylene biosynthesis, which might counteract the miRNA-triggered activation of ERF genes and thus the activation of defense pathways. At 30 dpi, ERF genes were upregulated, which is consistent with increased defense responses. Further investigation is required to validate an interaction between ethylene and the miRNA-mediated growth–defense tradeoff.

In TC48 at 30 dpi, showed decreased levels of miR397, which targets multiple LAC genes (Fig. 2E). Overexpressing *MIR397* in Arabidopsis decreased the expression of *AtLAC2/4/17* and lignin content, whereas downregulating *MIR397* increased *LAC2* expression [17]. Therefore, at 30 dpi, multiple LAC genes in TC48 are likely upregulated due to the reduced abundance of miR397. ST-8, however, had decreased levels of miRNAs targeting TCP family transcription factor genes at 30 dpi, suggesting differential transcriptional reprogramming (Fig. 2F), but increased levels of other miRNAs, including miR396, which targets genes encoding growth-regulating factors (GRFs) (Fig. 2G). Changes in the abundance several pre-miRNAs and transcripts of their target genes were validated by RT-qPCR (Fig. S3B–D). We performed GO analysis of these de-miRNA target genes. Genes involved in various metabolic pathways were upregulated in TC48 at 7 dpi and downregulated at 30 dpi, which is consistent with the bimodal dynamic energy metabolism described above (Fig. 2H). Together, our observations suggest that miRNAs likely participate in the fine-tuning of the defense–growth tradeoff in both TC48 and ST-8 in response to CMV infection. Please note that these predictions about miRNAs and their target genes are solely based on bioinformatic analysis. Further experiments are needed to validate the miRNA–targets relationships.

We next investigated the potential involvement of 24-nt siRNAs in the CMV responses of TC48. We defined de-siRNAs based on the following criteria (Fig. S4A): (1) Log_2_(fold change) in CPM >1; and (2) the region contains CG methylation >0.5, CHG methylation >0.4, and CHH methylation >0.2, defining heterochromatic regions where 24-nt siRNAs are commonly generated. As 24-nt siRNAs repress transcription, we identified genes located within 1 kb downstream of the de-siRNAs and ranked them by proximity. However, we did not observe significant changes in the expression of these genes in response to CMV at either 7 dpi or 30 dpi (Fig. 2I; Fig. S4A and B). Therefore, under our experimental conditions and analysis pipeline, these de-siRNAs did not appear to significantly influence the expression of their downstream proximal genes. However, this conclusion is based on unbiased bulk analysis. We cannot exclude the possibility that siRNAs might regulate the expression of these genes in certain individuals, which would not be reflected by bulk analysis.

To identify quantitative trait loci (QTLs) underlying the CMV responses of TC48, we crossed TC48 to ST-8 and obtained an F2 population for BSA (Supplementary Methods). F1 hybrids of TC48 and ST-8 displayed intermediate resistance to CMV at 30 dpi compared to TC48 and ST-8, indicating that it is unlikely that this resistance is conferred by a dominant allele (Fig. S5A). The F2 population displayed a continuous range of resistance phenotypes that did not conform to a 1:2:1 segregation ratio, suggesting that this trait is likely controlled by QTLs (Fig. S5B and C). For BSA, we selected the 18 most resistant (R) and 23 most sensitive (S) individuals from the F2 population and performed whole-genome resequencing on the R pool, S pool, and the parental line TC48. We calculated the delta SNP index by comparing the R pool with the S pool and the S pool with TC48. Genomic regions identified in both comparisons were considered to be candidate regions conferring the CMV response in TC48. This approach revealed regions on chromosomes Chr01, Chr03, Chr06, Chr07, and Chr10 that were linked to the resistant phenotype (Fig. S5D). To narrow down the region identified on Chr07, we developed derived cleaved amplified polymorphic sequence (dCAPS) markers, which defined a candidate interval spanning 20.3 Mb to 162 Mb on Chr07 (Fig. S5E and F). No additional recombination events within this region were identified in our population. Annotated genes harboring nucleotide sequence polymorphisms, including SNPs and InDels, are listed in Table S1.

Due to the limited size of the F2 population, we were unable to further narrow down the candidate regions. Additionally, the recombination rate was particularly low within certain candidate regions, such as the one identified on Chr 07, likely due to its proximity to the centromere. To further narrow down the regions and identify the causal genes, a significantly larger mapping population is needed. Functional validation by generating CRISPR knockout mutants or transgenic overexpressing lines in pepper remains challenging. Alternatively, functional confirmation could be performed in its close relative tomato (*Solanum lycopersicum*).

Using the mapping population, we evaluated two additional phenotypes of ST-8 and TC48 after CMV infection (Fig. S6A): plant height and leaf chlorosis. Leaf chlorosis was measured as previously described [21,22]. ST-8 showed reduced plant height after CMV infection while the plant height of TC48 remained unchanged. The resistant pool was significantly taller than the sensitive pool after CMV infection (Fig. S6B). Similarly, ST-8 displayed obvious leaf chlorosis while TC48 leaves remained green. The sensitive pool showed more obvious leaf chlorosis than the resistant pool (Fig. S6C).

In summary, TC48 exhibited minimal disease symptoms during both vegetative and reproductive growth after CMV infection. In the future, additional physiological fitness measurements should be performed to further validate this tolerance to CMV. At the transcriptional level, we observed bimodal dynamics of photosynthesis and defense responses at different stages of infection and showed that miRNAs are likely involved in this regulation. We propose that the critical difference in CMV tolerance between TC48 and ST-8 occurs at the earlier stages of infection, when CMV is not systemically spreading. At this time, photosynthesis is transcriptionally activated in TC48 systemic leaves, whereas defense responses are transcriptionally activated in ST-8 systemic leaves. We hypothesize that these early transcriptomic differences might lead to energy allocation that better supports growth in TC48, leading to healthier plants when CMV later spreads systemically. While the genetic determinants of the CMV responses in TC48 and a comprehensive understanding of these responses await further investigation, TC48 represents a valuable genetic resource for improving the responses of pepper to CMV infection. Our findings provide much-needed mechanistic insights into the molecular mechanisms behind CMV responses.

## CRediT authorship contribution statement

**Tonghui Li:** Writing – review & editing, Visualization, Investigation, Formal analysis, Data curation. **Xi Wang:** Writing – review & editing, Investigation, Data curation. **Xueyan Yao:** Writing – review & editing, Investigation, Funding acquisition. **Ye Liu:** Investigation. **Manru Song:** Investigation. **Yingjie Mi:** Formal analysis. **Hongji Yang:** Formal analysis. **Lulu Liu:** Investigation. **Enguang Li:** Investigation. **Junyue Sun:** Investigation. **Hailong Yu:** Conceptualization. **Nir Ohad:** Conceptualization. **Xuan Huang:** Conceptualization. **Feng Liu:** Conceptualization. **Yan Xue:** Writing – original draft, Supervision, Conceptualization.

## Declaration of competing interest

The authors declare that they have no known competing financial interests or personal relationships that could have appeared to influence the work reported in this paper.

## Data Availability

Data supporting the findings of this work are available within the paper and its Supplementary Information files. The datasets generated and analyzed during the current study are available from the corresponding author upon request. All high-throughput sequencing data generated in this study are accessible at Genome Sequence Archive (GSA) under accession number CRA038365 (https://ngdc.cncb.ac.cn/gsa/s/i0lL1r8m). Before publication, the data will be accessible only via the private reviewer link: https://ngdc.cncb.ac.cn/gsa/s/i0lL1r8m. The dataset includes RNA seq (CRR2704772–CRR2704795), small RNA seq (CRR2704796–CRR2704815), and BSA whole-genome resequencing (CRR3171676–CRR3171678) data.
