# Supplementary material for: Capsicum annuum TC48 shows both resistance and tolerance to cucumber mosaic virus and is a potential genetic resource for pepper improvement
Source: aBIOTECH. 2026 Jul 29;7(4):100081. doi: 10.1016/j.abiote.2026.100081 (PMC13562080; doi:10.1016/j.abiote.2026.100081)
Supplement: Supplementary file 1 [file mmc10.pdf]

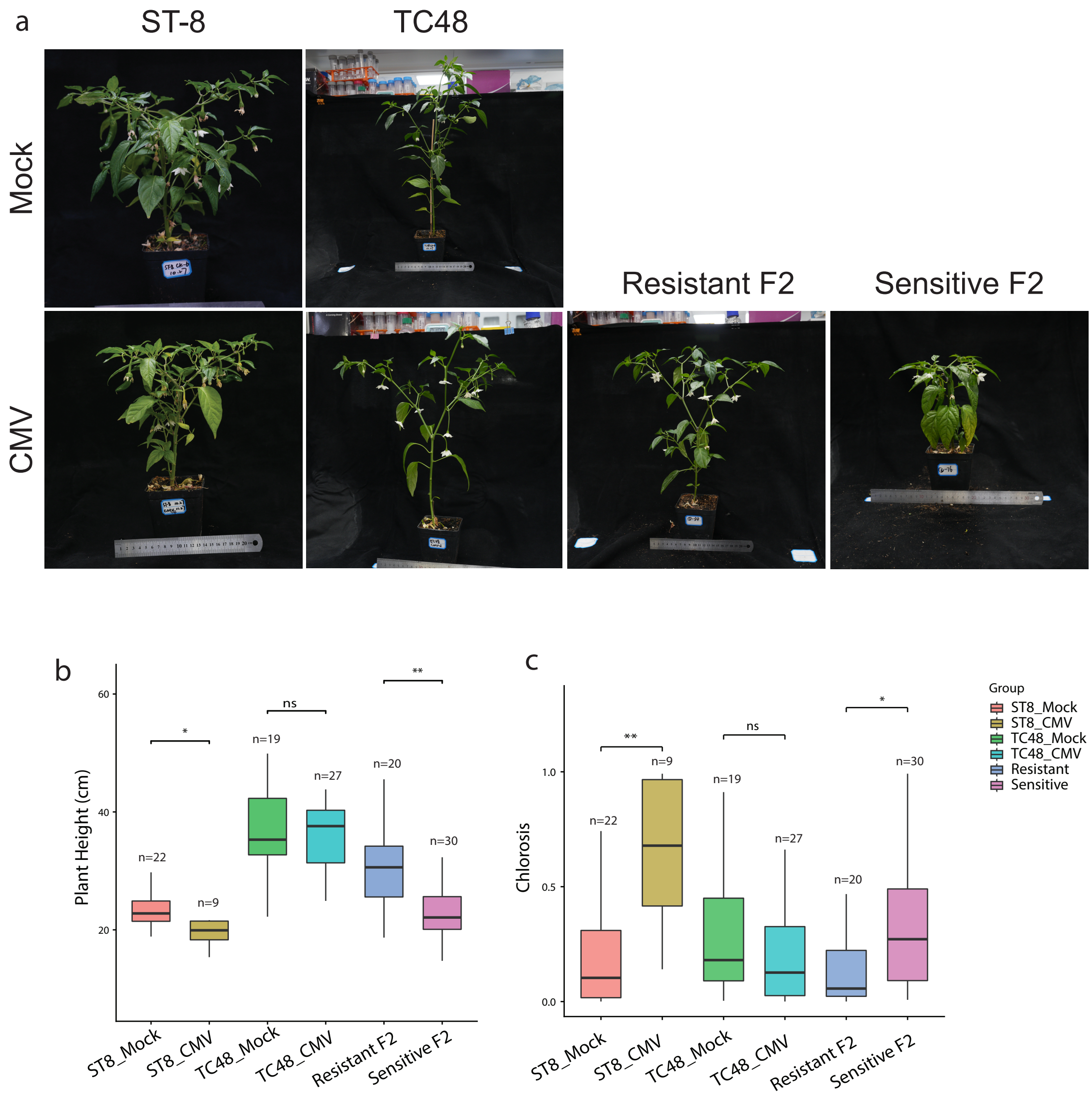
a
ST-8
TC48
Mock
CMV
Resistant F2
Sensitive F2
b
Plant Height (cm)
60
40
20
*
ns
**
n=22
n=9
n=19
n=27
n=20
n=30
ST8_Mock
ST8_CMV
TC48_Mock
TC48_CMV
Resistant F2
Sensitive F2
c
Chlorosis
1.0
0.5
0.0
**
ns
*
n=22
n=9
n=19
n=27
n=20
n=30
ST8_Mock
ST8_CMV
TC48_Mock
TC48_CMV
Resistant F2
Sensitive F2
Group
ST8_Mock
ST8_CMV
TC48_Mock
TC48_CMV
Resistant
Sensitive
