# Supplementary material for: Capsicum annuum TC48 shows both resistance and tolerance to cucumber mosaic virus and is a potential genetic resource for pepper improvement
Source: aBIOTECH. 2026 Jul 29;7(4):100081. doi: 10.1016/j.abiote.2026.100081 (PMC13562080; doi:10.1016/j.abiote.2026.100081)
Supplement: Supplementary file 2 [file mmc5.pdf]

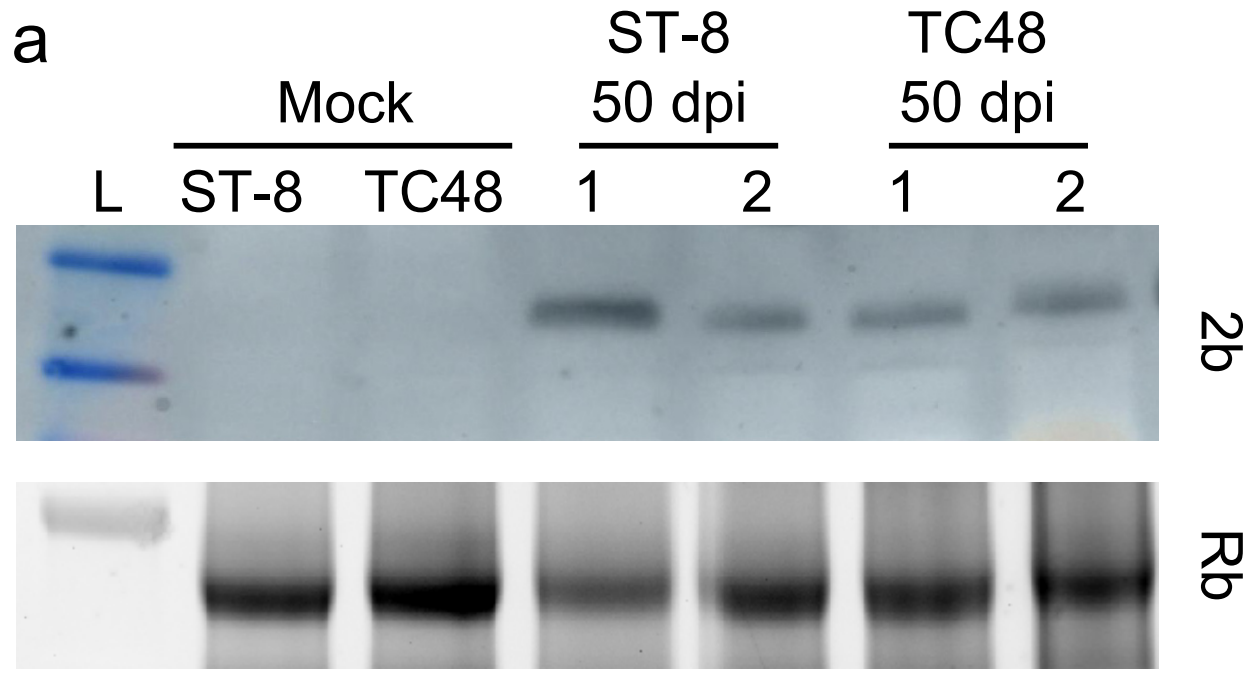
a
Mock
ST-8
50 dpi
TC48
50 dpi
L
ST-8
TC48
1
2
1
2
2b
Rb
