# Supplementary material for: Capsicum annuum TC48 shows both resistance and tolerance to cucumber mosaic virus and is a potential genetic resource for pepper improvement
Source: aBIOTECH. 2026 Jul 29;7(4):100081. doi: 10.1016/j.abiote.2026.100081 (PMC13562080; doi:10.1016/j.abiote.2026.100081)
Supplement: Supplementary file 3 [file mmc6.pdf]

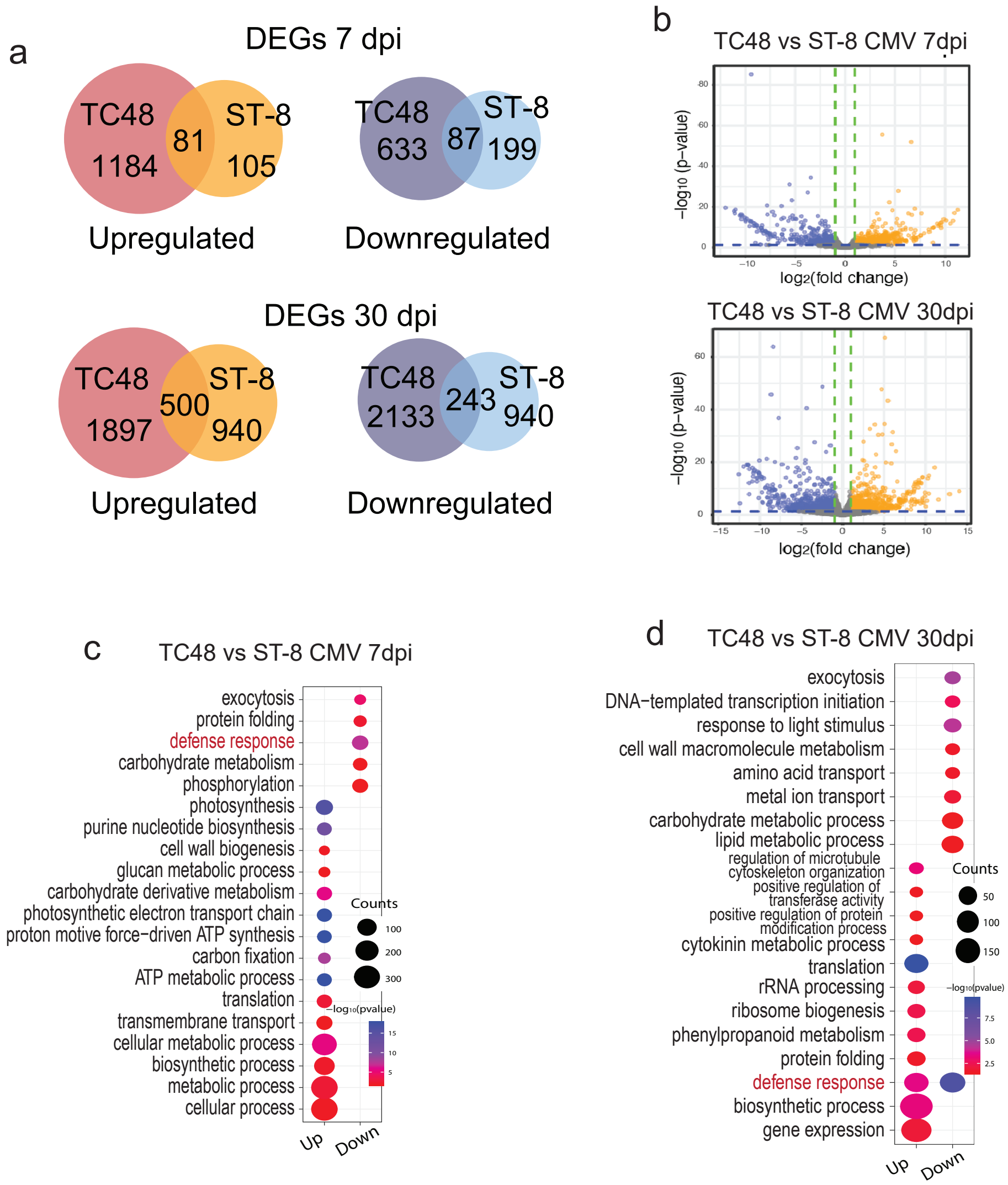

a
DEGs 7 dpi
TC48 1184
81
ST-8 105
Upregulated
TC48 633
87
ST-8 199
Downregulated
DEGs 30 dpi
TC48 1897
500
ST-8 940
Upregulated
TC48 2133
243
ST-8 940
Downregulated
b
TC48 vs ST-8 CMV 7dpi
−log10 (p−value)
log2(fold change)
TC48 vs ST-8 CMV 30dpi
−log10 (p−value)
log2(fold change)
c
TC48 vs ST-8 CMV 7dpi
exocytosis
protein folding
defense response
carbohydrate metabolism
phosphorylation
photosynthesis
purine nucleotide biosynthesis
cell wall biogenesis
glucan metabolic process
carbohydrate derivative metabolism
photosynthetic electron transport chain
proton motive force−driven ATP synthesis
carbon fixation
ATP metabolic process
translation
transmembrane transport
cellular metabolic process
biosynthetic process
metabolic process
cellular process
Counts
100
200
300
−log10(pvalue)
15
10
5
Up
Down
d
TC48 vs ST-8 CMV 30dpi
exocytosis
DNA−templated transcription initiation
response to light stimulus
cell wall macromolecule metabolism
amino acid transport
metal ion transport
carbohydrate metabolic process
lipid metabolic process
regulation of microtubule cytoskeleton organization
positive regulation of transferase activity
positive regulation of protein modification process
cytokinin metabolic process
translation
rRNA processing
ribosome biogenesis
phenylpropanoid metabolism
protein folding
defense response
biosynthetic process
gene expression
Counts
50
100
150
−log10(pvalue)
7.5
5.0
2.5
Up
Down
