# Supplementary material for: Capsicum annuum TC48 shows both resistance and tolerance to cucumber mosaic virus and is a potential genetic resource for pepper improvement
Source: aBIOTECH. 2026 Jul 29;7(4):100081. doi: 10.1016/j.abiote.2026.100081 (PMC13562080; doi:10.1016/j.abiote.2026.100081)
Supplement: Supplementary file 4 [file mmc7.pdf]

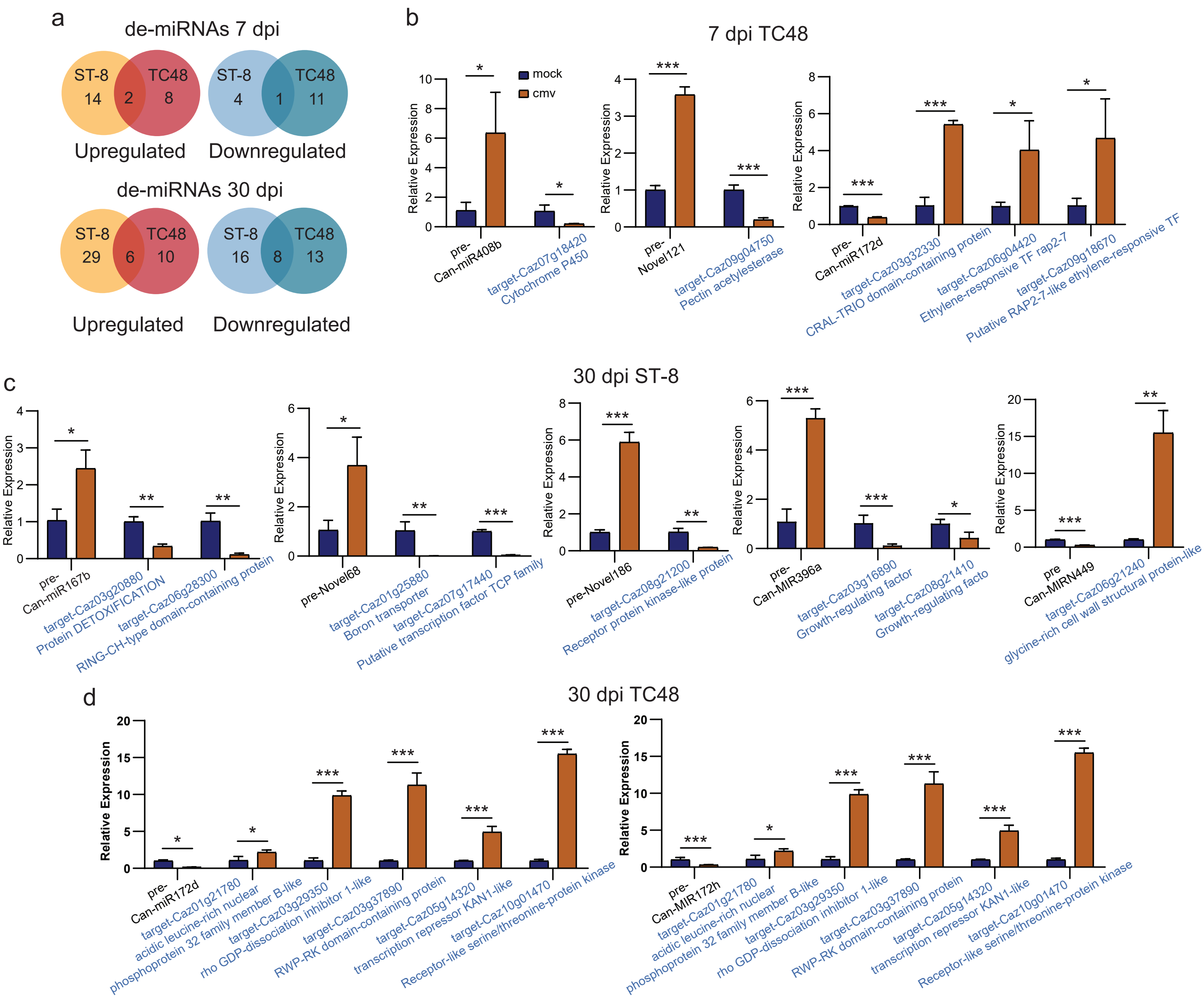

a
de-miRNAs 7 dpi
ST-8 14 2 TC48 8
Upregulated
ST-8 4 1 TC48 11
Downregulated
de-miRNAs 30 dpi
ST-8 29 6 TC48 10
Upregulated
ST-8 16 8 TC48 13
Downregulated
b
7 dpi TC48
mock
cmv
Relative Expression
pre-Can-miR408b
target-Caz07g18420 Cytochrome P450
pre-Novel121
target-Caz09g04750 Pectin acetylesterase
pre-Can-miR172d
target-Caz03g32330 CRAL-TRIO domain-containing protein
target-Caz06g04420 Ethylene-responsive TF rap2-7
target-Caz09g18670 Putative RAP2-7-like ethylene-responsive TF
c
30 dpi ST-8
pre-Can-miR167b
target-Caz03g20880 Protein DETOXIFICATION
target-Caz06g28300 RING-CH-type domain-containing protein
pre-Novel68
target-Caz01g25880 Boron transporter
target-Caz07g17440 Putative transcription factor TCP family
pre-Novel186
target-Caz08g21200 Receptor protein kinase-like protein
pre-Can-MIR396a
target-Caz03g16890 Growth-regulating factor
target-Caz08g21410 Growth-regulating facto
pre Can-MIRN449
target-Caz06g21240 glycine-rich cell wall structural protein-like
d
30 dpi TC48
pre-Can-miR172d
pre-Can-MIR172h
target-Caz01g21780 acidic leucine-rich nuclear phosphoprotein 32 family member B-like
target-Caz03g29350 rho GDP-dissociation inhibitor 1-like
target-Caz03g37890 RWP-RK domain-containing protein
target-Caz05g14320 transcription repressor KAN1-like
target-Caz10g01470 Receptor-like serine/threonine-protein kinase
