# Supplementary material for: Capsicum annuum TC48 shows both resistance and tolerance to cucumber mosaic virus and is a potential genetic resource for pepper improvement
Source: aBIOTECH. 2026 Jul 29;7(4):100081. doi: 10.1016/j.abiote.2026.100081 (PMC13562080; doi:10.1016/j.abiote.2026.100081)
Supplement: Supplementary file 5 [file mmc8.pdf]

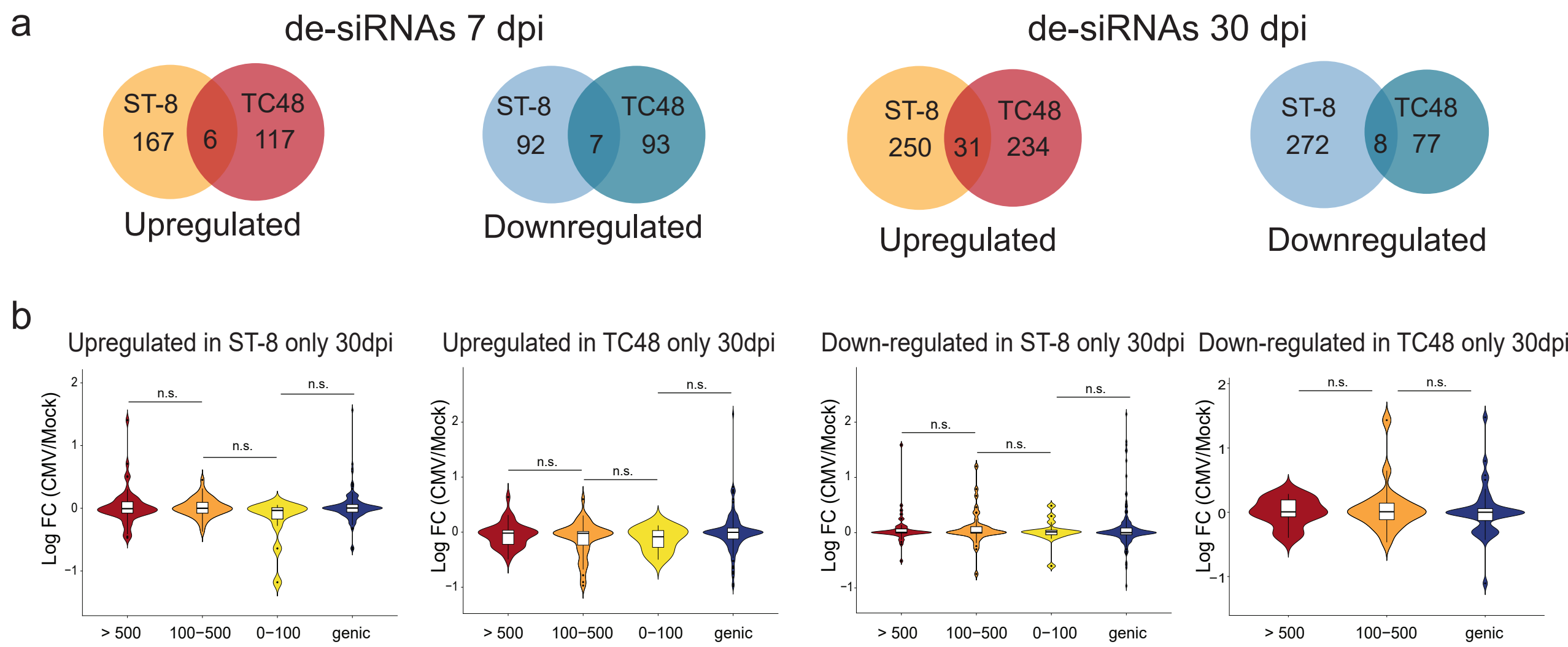
a
de-siRNAs 7 dpi
de-siRNAs 30 dpi
ST-8
167
6
TC48
117
Upregulated
ST-8
92
7
TC48
93
Downregulated
ST-8
250
31
TC48
234
Upregulated
ST-8
272
8
TC48
77
Downregulated
b
Upregulated in ST-8 only 30dpi
Upregulated in TC48 only 30dpi
Down-regulated in ST-8 only 30dpi
Down-regulated in TC48 only 30dpi
Log FC (CMV/Mock)
n.s.
> 500
100−500
0−100
genic
