# Supplementary material for: Capsicum annuum TC48 shows both resistance and tolerance to cucumber mosaic virus and is a potential genetic resource for pepper improvement
Source: aBIOTECH. 2026 Jul 29;7(4):100081. doi: 10.1016/j.abiote.2026.100081 (PMC13562080; doi:10.1016/j.abiote.2026.100081)

a

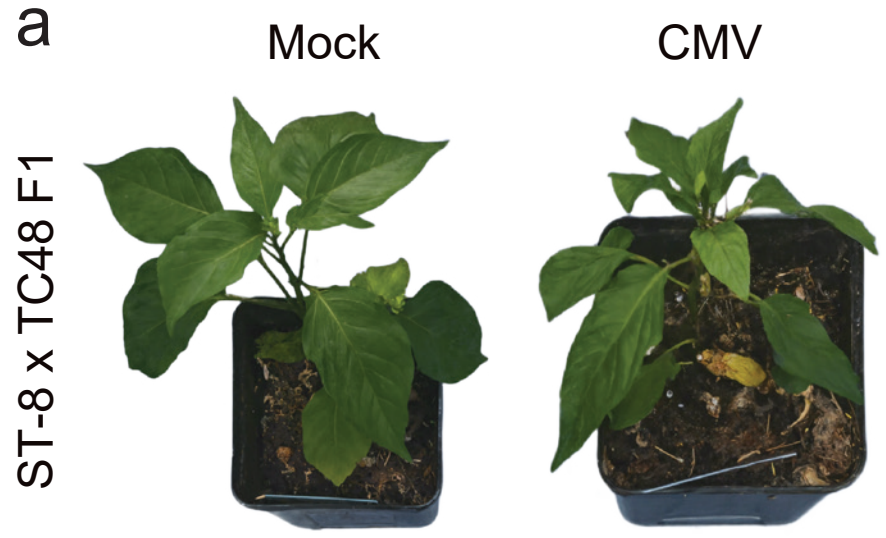

b

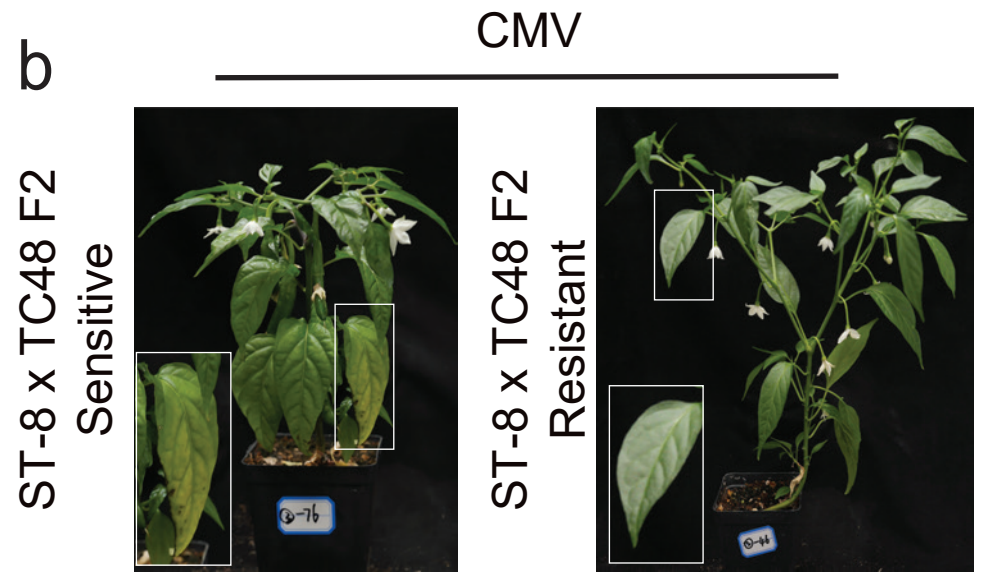

c

Population	Number of Plants: Resistant (DI = 1 to 2)	Intermediate (DI = 2 to 4)	Susceptible (DI = 4 to 5)	Total	Segregating Ratio	Chi-Square Value	Probability Value
F2 (ST-8xTC48)	66	180	41	287	1:2:1	22.923	1.053e-05

d

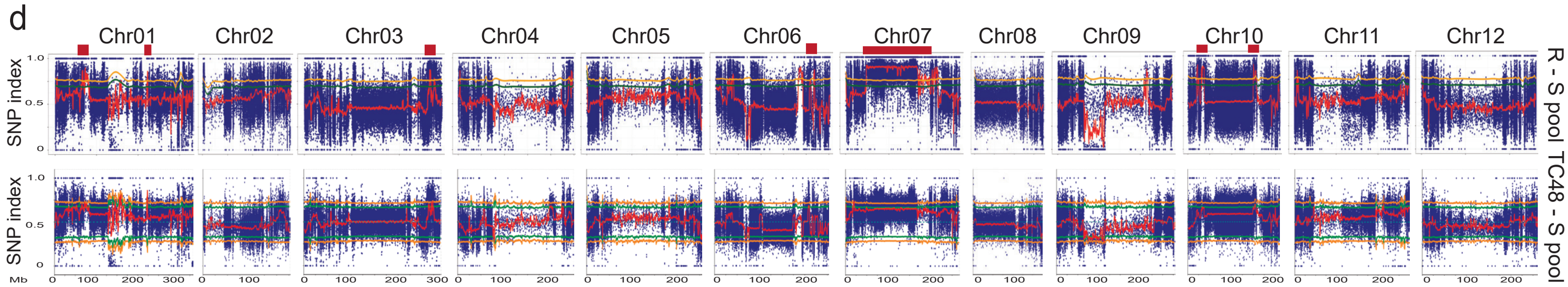

e

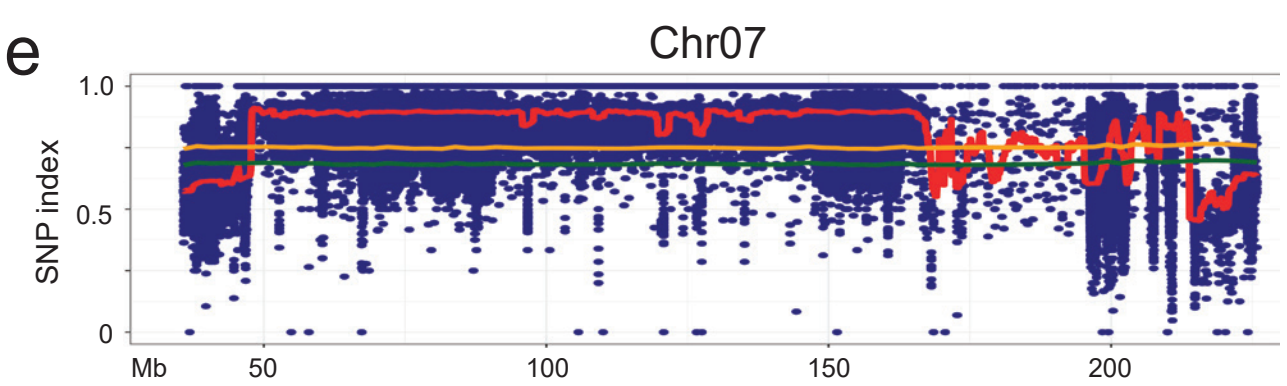

f

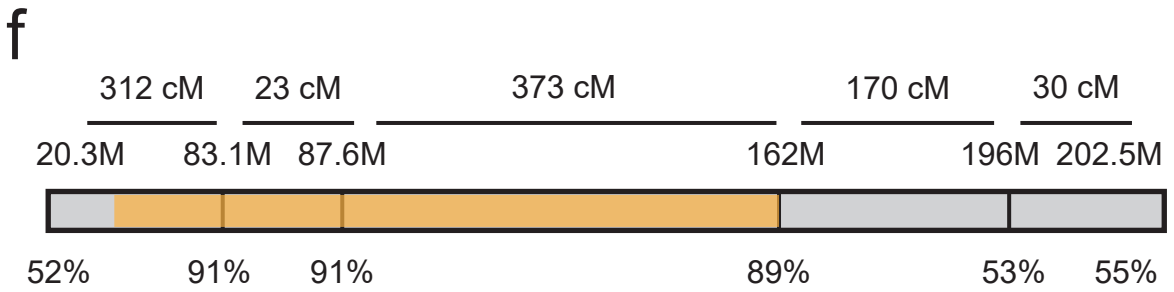

Supplement: Supplementary file 6 [file mmc9.pdf]
