# Supplementary material for: Capsicum annuum TC48 shows both resistance and tolerance to cucumber mosaic virus and is a potential genetic resource for pepper improvement
Source: aBIOTECH. 2026 Jul 29;7(4):100081. doi: 10.1016/j.abiote.2026.100081 (PMC13562080; doi:10.1016/j.abiote.2026.100081)
Supplement: Supplementary file 7 [file mmc11.docx]

**1. Materials and methods**

*1.1. Plant materials and growth conditions*

*C. annuum* lines ST-8 and TC48 were kindly provided by Dr. Feng Liu. The seeds were germinated at 28 ^°^C in the dark, transferred to soil, and cultured in a greenhouse under long-day conditions (16 h light/8 h dark at 28 ^°^C).

*1.2. Viral inoculation and evaluation of disease symptoms*

CMV_FNY_ was kindly provided by Dr. Qian Zhou and was propagated on tobacco (*N. tabacum*). The virus inoculum was prepared by grinding 1 g of infected tobacco leaves in 5 mL of 10 mM phosphate buffer (pH 7.0), filtered through two layers of cloth, and injected into the first two true leaves of pepper seedlings at the 6-leaf stage. The inoculated seedlings were maintained in a greenhouse. Disease symptoms were monitored at 30 dpi.

Disease severity was evaluated based on the degree of mosaic patterning and leaf distortion and indicated as the Disease Index (DI) [1]. DI=1: no symptoms; DI=1.5: a few leaves display mild distortion; DI=2: a few leaves display intermediate distortion and mild mosaic patterning; DI=2.5: less than half of the leaves display intermediate distortion and mild mosaic patterning; DI=3: more than half of the leaves display intermediate distortion and mild mosaic patterning; DI=3.5: less than half of the leaves display severe distortion and mosaic patterning; DI=4: more than half of the leaves display severe distortion and mosaic patterning; DI=4.5: almost all leaves display severe distortion and mosaic patterning; DI=5: all leaves display severe distortion and mosaic patterning. Evaluations were performed by 5 independent individuals, and the mean values were taken as the DI.

*1.3. Protein quantification*

Discs from two systemic leaves were pooled at each time point. Immunoblotting was performed using anti-2b primary antibody (Agisera) and HRP-conjugated goat anti-rabbit secondary antibody (ABclonal). Detection was performed using SuperSignal^TM^ West Atto substrate (Thermo Fisher Scientific). Rubisco was visualized directly in SDS-PAGE gels (Epizyme) under UV exposure. Band intensity was quantified with ImageJ, and the quantity of 2b was calculated as intensity of 2b/Rubisco.

*1.4. RNA seq*

RNA-seq was performed with three biological replicates. Each biological replicate contained pooled samples from three plants. Total RNA was extracted from the samples using FreeZol Reagent according to the manufacturer’s instruction (Vazyme). Libraries were constructed using a VAHTS Unive V8 RNA-seq Library Prep Kit for Illumina according to the manufacturer’s instructions (Vazyme). Sequencing was performed on an Illumina NovaSeq 6000 system.

Reads were mapped to the ST-8 reference genome using HISAT2 (v2.2.1)[2]. Expression abundance was calculated using the Rsubread package (v2.14.1)[3] and Trinity (v2.8.5). The gene expression data were normalized using TPM (transcripts per million). DEGs were called using DEseq2 [4]. GO analysis was performed with TBtools-II [5].

*1.5. sRNA seq*

Total RNA was extracted from the samples using FreeZol Reagent (Vazyme). One microgram of total RNA was used to prepare a small RNA library with a VAHTS Small RNA Library Prep Kit for Illumina (Vazyme) according to the manufacturer’s instructions. The small RNA library was sequenced on the Illumina NovaSeq 6000 platform.

For miRNA analysis, annotated pre-miRNAs for *Capsicum annuum* deposited in miRBase release 22 were mapped to the ST-8 reference genome using Bowtie, keeping only uniquely mapped loci with the highest alignment scores [6, 7]. sRNA seq reads 21- and 22-nt in length were then mapped to the annotated pre-miRNAs using Bowtie to identify known miRNAs. To identify novel miRNAs, non-coding RNAs were discarded, including rRNAs, tRNAs, snRNAs, and snoRNAs [8]. Repeat sequences in the ST-8 genome were then annotated using RepeatMasker, and sRNA sequences that mapped to the repeats were discarded (http://www.repeatmasker.org). The remaining reads were mapped to the annotated ST-8 genome. Among these, reads annotated as “unknown” or “intron” were retained for novel miRNA prediction using miRDeep-P2 (v1.1.4), which predicts both mature miRNAs and their corresponding precursors [9]. De-miRNAs and target gene pairs were defined based on *p*-value < 0.01 and |log_2_FC| >1, with fold-change (FC) values in the opposite direction.

For siRNA analysis, reads 24-nt in length were retained. To identify RdDM sites, where 24-nt siRNAs are produced, only regions with mCG > 0.5, mCHG > 0.4 and mCHH > 0.2 were retained for further analysis. de-siRNA regions were called with MACS2 [10], and regions with FC > 2 were retained. de-siRNA proximal genes were identified using ChIPseeker [11].

*1.6. Bulk segregant analysis*

To conduct BSA, TC48 was crossed with ST-8. F1 and F2 individuals were infected with CMV, and disease susceptibility was evaluated. A total of 297 F2 plants were phenotyped. The 18 most susceptible and 23 most resistant individuals were selected. DNA from these 41 individuals was extracted and pooled equally into the R and S pools, which were subjected to whole genome resequencing at 50x coverage each with the Illumina NovaSeq X Plus system. The sequencing data were quality controlled and mapped to the ST-8 reference genome. SNPs were called using GATK, and the delta SNP index was calculated using QTL-seq software.

*1.7 qRT-PCR*

qRT-PCR was performed on a Bio-Rad CFX96 real-time PCR system using 2× SYBR Green qPCR Mix (SparkJade, AH0104). Melting curve analysis was conducted to verify the specificity of amplification. Each reaction was performed in three technical replicates, and relative expression levels were calculated using the 2^-ΔΔCt^ method, normalized to the internal reference gene *UBI3* (ubiquitin-conjugating protein).

[1]	Guo G, Wang S, Liu J, Pan B, Diao W, Ge W, et al. Rapid identification of QTLs underlying resistance to cucumber mosaic virus in pepper (*Capsicum frutescens*). Theor Appl Genet 2017;130(1):41-52.

[2]	Kim D, Paggi JM, Park C, Bennett C, Salzberg SL. Graph-based genome alignment and genotyping with HISAT2 and HISAT-genotype. Nat Biotechnol 2019;37(8):907-15.

[3]	Liao Y, Smyth GK, Shi W. The R package rsubread is easier, faster, cheaper and better for alignment and quantification of RNA sequencing reads. Nucleic Acids Res 2019;47(8):e47.

[4]	Lataretu M, Holzer M. RNAflow: an effective and simple RNA-Seq differential gene expression pipeline using nextflow. Genes (Basel) 2020;11(12).

[5]	Chen C, Wu Y, Li J, Wang X, Zeng Z, Xu J, et al. TBtools-II: a "one for all, all for one" bioinformatics platform for biological big-data mining. Mol Plant 2023;16(11):1733-42.

[6]	Langmead B, Trapnell C, Pop M, Salzberg SL. Ultrafast and memory-efficient alignment of short DNA sequences to the human genome. Genome Biol 2009;10(3):R25.

[7]	Kozomara A, Birgaoanu M, Griffiths-Jones S. miRBase: from microRNA sequences to function. Nucleic Acids Res 2019;47(D1):D155-D62.

[8]	Kalvari I, Nawrocki EP, Ontiveros-Palacios N, Argasinska J, Lamkiewicz K, Marz M, et al. Rfam 14: expanded coverage of metagenomic, viral and microRNA families. Nucleic Acids Res 2021;49(D1):D192-D200.

[9]	Kuang Z, Wang Y, Li L, Yang X. miRDeep-P2: accurate and fast analysis of the microRNA transcriptome in plants. Bioinformatics 2019;35(14):2521-2.

[10]	Feng J, Liu T, Qin B, Zhang Y, Liu XS. Identifying ChIP-seq enrichment using MACS. Nat Protoc 2012;7(9):1728-40.

[11]	Yu G, Wang LG, He QY. ChIPseeker: an R/Bioconductor package for ChIP peak annotation, comparison and visualization. Bioinformatics 2015;31(14):2382-3.
