# Supplementary material for: Capsicum annuum TC48 shows both resistance and tolerance to cucumber mosaic virus and is a potential genetic resource for pepper improvement
Source: aBIOTECH. 2026 Jul 29;7(4):100081. doi: 10.1016/j.abiote.2026.100081 (PMC13562080; doi:10.1016/j.abiote.2026.100081)
Supplement: Supplementary file 8 [file mmc4.docx]

**Supplementary Figure Legends**

**Supplementary Figure 1 TC48 display partial resistance and tolerance to CMV. a** Anti-2b western blot with mock treated ST-8 or TC48 at 50 dpi.

**Supplementary Figure 2 Transcriptional reprogramming underlying CMV resistance in TC48. a** Venn diagrams of up- and down-regulated genes in TC48 relative to ST-8 7 dpi and 30 dpi of CMV infection. **b** Vocano plots showing the DEGs in TC48 after CMV inoculation compared to ST-8. **c-d** GO enrichment of DEGs in TC48 compared to ST-8 7 dpi (**c**) or 30 dpi (**d**) of CMV inoculation.

**Supplementary Figure 3 Transcriptional reprogramming underlying CMV resistance in TC48. a** Venn diagrams showing the overlap between de-miRNAs in ST-8 and TC48 7 dpi and 30 dpi of CMV infection. **b-d** Validation of pre-miRNAs and their target gene expression changes in ST-8 and TC48 at 7 dpi and 30 dpi by RT-qPCR.

**Supplementary Figure 4 Functions of de-siRNA. a** Venn diagrams showing the overlap of de-siRNAs in ST-8 and TC48 at 7 and 30 dpi of CMV. **b** Log FC of expression in CMV/Mock of genes down-stream of the de-siRNAs 30 dpi of CMV.

**Supplementary Figure 5 Genetic basis of TC48 CMV responses. a** Representative images of ST-8×TC48 F1 hybrid with mock or CMV inoculation. **b** Representative images of a sensitive and a resistant individual in the F2 population. **c** Segregation ratio of resistance in the F2 population. **d** Delta-SNP index plot by comparing the R pool to the S pool and the TC48 to the S pool. **e** Magnified view of a candidate region on chromosome 7. **f** Recombination rate of 6 dCAPS markers within the candidate region on chromosome 7.

**Supplementary Figure 6 Measurements of plant hight and leaf chlorosis in ST-8, TC48, the resistant and sensitive F2 pools. a** Representative phenotypes of the parental lines, resistant F2 and sensitive F2 after CMV inoculation.The image of the sensitive F2 plant inoculated with CMV in panel a is also shown in Supplementary Figure 5b. b-c Height (b) and leaf chlorosis levels (c) of the indicated plants after mock and CMV inoculation.
